# Enhanced Neuronal Glucose Transporter Expression Reveals Metabolic Choice in a HD *Drosophila* Model

**DOI:** 10.1371/journal.pone.0118765

**Published:** 2015-03-11

**Authors:** Marie Thérèse Besson, Karin Alegría, Pamela Garrido-Gerter, Luis Felipe Barros, Jean-Charles Liévens

**Affiliations:** 1 Aix-Marseille Université, CNRS, CRN2M-UMR7286, 13344 Marseille cedex 15, Marseille, France; 2 Centro de Estudios Científicos, Arturo Prat 514, Valdivia, Chile; 3 Universidad Austral de Chile, Valdivia, Chile; National Center for Geriatrics and Gerontology, JAPAN

## Abstract

Huntington’s disease is a neurodegenerative disorder caused by toxic insertions of polyglutamine residues in the Huntingtin protein and characterized by progressive deterioration of cognitive and motor functions. Altered brain glucose metabolism has long been suggested and a possible link has been proposed in HD. However, the precise function of glucose transporters was not yet determined. Here, we report the effects of the specifically-neuronal human glucose transporter expression in neurons of a *Drosophila* model carrying the exon 1 of the human *huntingtin* gene with 93 glutamine repeats (HQ93). We demonstrated that overexpression of the human glucose transporter in neurons ameliorated significantly the status of HD flies by increasing their lifespan, reducing their locomotor deficits and rescuing eye neurodegeneration. Then, we investigated whether increasing the major pathways of glucose catabolism, glycolysis and pentose-phosphate pathway (PPP) impacts HD. To mimic increased glycolytic flux, we overexpressed phosphofructokinase (PFK) which catalyzes an irreversible step in glycolysis. Overexpression of PFK did not affect HQ93 fly survival, but protected from photoreceptor loss. Overexpression of glucose-6-phosphate dehydrogenase (G6PD), the key enzyme of the PPP, extended significantly the lifespan of HD flies and rescued eye neurodegeneration. Since G6PD is able to synthesize NADPH involved in cell survival by maintenance of the redox state, we showed that tolerance to experimental oxidative stress was enhanced in flies co-expressing HQ93 and G6PD. Additionally overexpressions of hGluT3, G6PD or PFK were able to circumvent mitochondrial deficits induced by specific silencing of genes necessary for mitochondrial homeostasis. Our study confirms the involvement of bioenergetic deficits in HD course; they can be rescued by specific expression of a glucose transporter in neurons. Finally, the PPP and, to a lesser extent, the glycolysis seem to mediate the hGluT3 protective effects, whereas, in addition, the PPP provides increased protection to oxidative stress.

## Introduction

Huntington’s disease (HD) is a heritable neurodegenerative disorder characterized by lesions in the striatum, progressive deterioration of motor and cognitive functions and psychiatric disturbances. HD is caused by expansion of a poly-glutamine (poly-Q) tract in the N-terminus of the Huntingtin protein (Htt) and consequently, accumulation of short N-terminus fragments of the protein. Poly-Q stretches of more than 36 residues are associated with pathology which affects the brain and numerous peripheral organs and tissues. New toxic functions were described for the mutated Htt; moreover, the mutation induces a loss of function of the wild-type Htt which plays an important role on cell survival and embryonic development [1]. Extensive therapeutic strategies were developed, but none of them proved to be effective in halting the disease progression and, up to date, treatments focus on alleviating HD-associated symptoms. Indeed, although the cascade of molecular and cellular events leading to mHtt pathology is still unclear, mHtt compromises several vital cellular functions such as intracellular trafficking [2], transcriptional regulation [3, 4], cytoskeleton [5], energy metabolism and mitochondrial functions [6], that were extensively described in reviews [7, 8].

Neurons depend on glucose for providing energy and redox protection. Because they are unable to synthesize or store glucose, neurons are fully dependent on glucose import. In human tissues, glucose homeostasis is mainly maintained by the members of the glucose transporter family (referred as SLC2A) comprising 14 isoforms mediating facilitative sugar transport [9]. The various isoforms show different affinity for glucose suggesting adaptation to the various metabolic requirements of each cell. In mammalian brain, although several GluT members are present, GluT1 and GluT3 are the predominant GluTs responsible for glucose transport [10]. GluT1 is detected in glial cells such as astrocytes [11, 12] and permits glucose storage into astrocytes by glycogen synthesis. GluT3 is the major neuronal GluT and transports glucose from the extracellular space into neurons [13, 14]. Glucose is metabolized through glycolysis in the cytosol, generating two molecules of pyruvate which fuel mitochondria where most ATP is produced. Neurons present relatively weak expression of glycolytic enzymes; in contrast, they favor another important metabolic pathway in glucose oxidation, the pentose phosphate pathway (PPP) in order to compensate for their limited antioxidant reserve [15, 16]. Indeed, the PPP provides intermediates for nucleotide synthesis and is the major source of cytosolic NADPH which is a critical factor for enzymes implied in cellular defense system against oxidative stress. The NADPH levels are mainly maintained by glucose 6-phosphate dehydrogenase (G6PD) activity, the first and rate-limiting enzyme of the PPP. As a by-product of energy production, the mitochondria also generate most of the endogenous reactive oxygen species (ROS), damaging both mitochondria and the rest of the cell. Thus, maintenance of mitochondrial integrity and function is the highest priority to brain cells. Any defect in brain mitochondria functioning may lead to severe energy deficiency as well as increased generation of ROS in neurons and ultimately to neuronal degeneration [17, 18, 19].

Numerous studies in HD patients and in animal models have indicated energy metabolism defects in the pathogenesis of HD before the occurrence of any overt pathology. They suggest that changes in energy metabolism are not a consequence of neuronal loss, but rather a contributory factor to the progression and development of the disease [20, 21, 22]. Studies on post-mortem brain tissue or positron emission tomography imaging analyses revealed reduced cerebral metabolic activity in cortex and striatum of symptomatic HD patients and also in pre-symptomatic subjects, so even before the onset of pathological symptoms [23, 24, 25, 26]. Moreover, Gamberino and Brennan [27] described decreases in glucose transporter levels by post-mortem analysis in caudate and not in cerebral cortex of HD brains; these decreases were not closely related to brain atrophy but can be associated to changes in transporter expressions. Interestingly, in a recent study on HD patients, a correlation was found between the onset of the disease and the copy number of the GluT3 gene [28]. Moreover, biochemical studies on different HD models tended to demonstrate dysfunctions linked to mitochondrial homeostasis [29]. The activity in oxidative phosphorylation was reduced [30, 31, 32]; they are associated with increased ROS levels and triggers oxidative impairment as observed in several neuropathological diseases [20, 33]. Plasma levels of oxidative damage products were found to be increased in HD patients and asymptomatic HD gene carriers [34]. Enzymes of the TCA cycle were impaired in post-mortem brain from HD patients or in HD models: mitochondrial aconitase presented a loss of activity [35] and pyruvate dehydrogenase (PDH) levels were altered in HD transgenic mice [36, 37].

*Drosophila* models for human neurodegenerative diseases (Alzheimer’s disease, Parkinson’s disease, or poly-Q diseases such as SCA1, HD…) have been created by expression of the relevant human pathogenic protein and provided many valuable insights into pathogenic mechanisms resulting in the course of hallmarks of these diseases [38, 39]. Specifically as in the HD, expression of the pathogenic Huntingtin in the *Drosophila* nervous system leads to neuropathology and premature cell death. The neuronal expression of the exon 1 of the human mutant huntingtin gene (containing 93 repeats of the CAG codon) in our *Drosophila* model (named hereafter HQ93 flies, or mHtt for mutated Huntingtin) affects functions of both neurons and glial cells [40]. Glucose homeostasis is maintained in a conserved manner in *Drosophila*, and metabolic regulation shows strong similarities to mammals [41,42].

Therefore, we postulated that impaired brain glucose metabolism in *Drosophila* contributes to HD pathogenesis and supposed altered expression of genes concerned with energy metabolism. In order to test these hypotheses, we generated transgenic *Drosophila* bearing the human glucose transporter hGluT3 and showed that its overexpression in HQ93 neurons is an effective approach to rescue lifespan, restore locomotor activity and slow down neurodegeneration. Results show that overexpressed PFK glycolytic enzyme in neurons has no impact on the early death of HQ93 flies; nevertheless it was able to decrease eye neurodegeneration, suggesting a moderate efficiency of increasing glycolysis. However, both organismal lifespan and neurodegeneration can be significantly rescued by overexpression of G6PD. As G6PD is involved in mechanisms of antioxidative defenses, we showed that resistance to experimental oxidative stress induced by H_2_O_2_ was enhanced in flies carrying both HQ93 and G6PD. In order to investigate the role of glucose metabolism on mitochondria defects which are one of the hallmarks of HD, we mimicked mitochondria dysfunction by silencing genes required for mitochondrial activities. Fly neurons were knocked down for genes expressed at the first step of the TCA cycle and in the first complex of the respiratory chain. We observed that hGluT3, PFK and G6PD transgenes increased the survival of flies presenting mitochondrial defects. This suggests that the mitochondrial activity deficit can be rescued by improving glucose metabolism. Finally, we conclude that increased neuronal glucose uptake by increasing glucose transporter expression alleviates HD pathogenesis. This effect was poorly mediated by up-regulation of glycolysis, but can be triggered by activation of G6PD in the PPP which permits to neurons to replenish NADPH pool and so, ameliorating the capacity of neurons to resist to oxidative stress induced by HQ93.

## Results

The transgenic *Drosophila* HD model used in this study has been widely exploited; it carried the exon 1 of the human mutated Huntingtin with 93 CAG repeats [40, 43]. In our experiments, expression of mHtt was regulated by the yeast UAS/Gal4 system in which the mHtt transgene lies downstream of the UAS sequence. HD flies were generated by crossing UAS-HQ93 flies to flies expressing the Gal4 protein in a tissue-specific manner. To achieve co-expression, crosses were conducted so that in the female F1 progeny, both UAS-mHtt and other UAS-transgenes were expressed in neurons under the regulation of the pan-neuronal specific *Elav*-Gal4 driver.

### Expression of a glucose transporter

**Fig 1:**
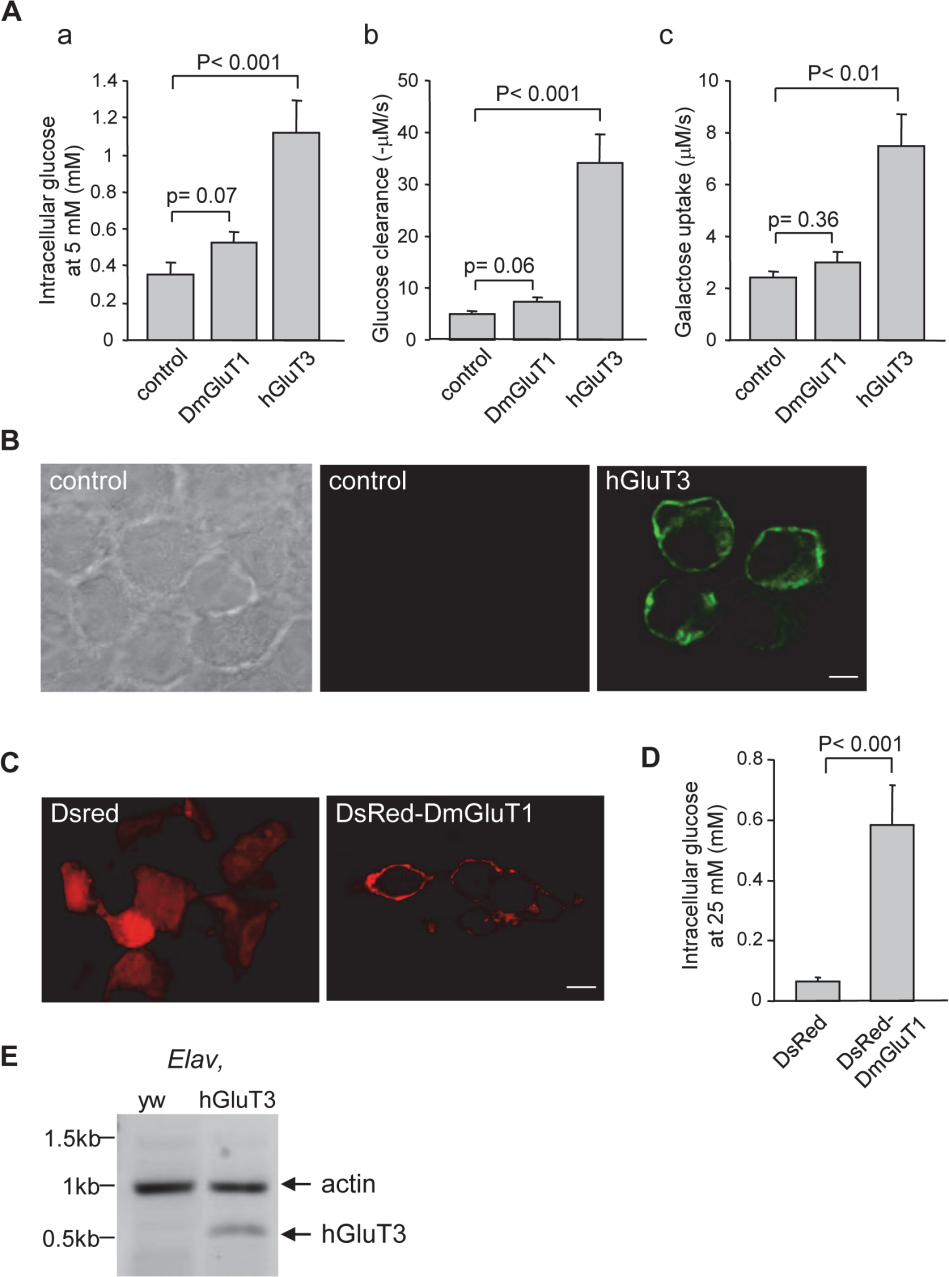
Expression and functional characterization of DmGluT1 and hGluT3. (**A**): Measurements of uptake parameters. HEK 293 cells were transfected with the glucose sensor and either an empty (control), *DmGluT1*, or *hGluT3* plasmids. Only *hGluT3* expression resulted in very significant increases in intracellular glucose concentration at 5mM extracellular glucose (a), glucose clearance (b) and galactose uptake (c). (**B**): *hGluT3* immunodetection: HEK-293 cells transfected with the empty plasmid were not labelled, with the *hGluT3* antibody (middle panel) whereas *hGluT3*-transfected cells revealed plasma membrane localization (right panel). The left panel shows *hGluT3*-transfected cells in bright field. Scale bar: 5 μm. (**C**): Detection of the *DsRed-DmGluT1* (right panel) showing its localization at the plasma membrane; The left panel shows *DsRed*-transfected cells as control. Scale bar: 5 μm. (**D**): Exposure of *DsRed*-*DmGlut1* cells to 25 mM extracellular glucose resulted in significantly higher intracellular glucose relative to control cells. (**E**): Detection of *hGluT3* and *actin* mRNA in flies expressing no transgene or *hGluT3* under the control of the *Elav-*Gal4 driver. RT-PCR analysis was performed from *Drosophila* heads at 4 days of adult age.

Previously we showed that the overexpression of an isoform of a predicted *Drosophila* sugar transporter family, referred as DmGluT1 [44], improved survival of flies expressing HQ93 in glial cells [45]. Since DmGluT1, like the other putative *Drosophila* orthologs of human sugar transporters, are not functionally characterized, we have verified whether DmGluT1 was able to transport glucose in comparison with the previously described activity of hGluT3 [46]. We measured the intracellular concentration of glucose with a FRET glucose sensor which allows estimation of transporter activity at cellular resolution in HEK-293 cells [47]. Using this method, we measured glucose in HEK-293 cells expressing the glucose sensor FLII^12^Pglu-700μΔ6 alone or in the presence of the DmGluT1 or hGluT3 cDNAs (Fig. 1A). GluT3 overexpression induced a strong significant increase in intracellular glucose concentration. In the presence of 5 mM extracellular glucose concentration, hGluT3-expressing cells maintained a steady-state intracellular glucose concentration averaging 1.12 + 0.17 mM instead of 0.35 + 0.03 mM for control cells. The S1 Fig. showed that hGluT3-expressing cells displayed cytoplasmic fluorescence for the glucose sensor compared to non-transfected cells. Glucose clearance was enhanced by hGluT3 expression after lowering extracellular glucose concentration to 0 mM (Fig. 1A). Finally, hGluT3-expressing cells also uptake galactose in accordance with previously characterized parameters in *Xenopus* ovocytes [46]. In the same conditions we found that DmGluT1 expression did not change glucose entry and clearance in HEK-293 cells, although close to significance levels (Fig. 1A); in contrast to hGluT3, no galactose uptake was observed with DmGluT1. In order to demonstrate that the low activity of DmGluT1 was not due to a failure of expression in HEK-293 cells, we also performed analysis using a DsRed-tagged construct. The localization of the tagged DmGluT1 was comparable to that of hGluT3 (Figs. 1B and 1C), namely at the plasma membrane. Nevertheless, DsRed-DmGluT1 expression was able to significantly increase intracellular glucose levels when HEK-293 cells were bathed with 25 mM extracellular glucose (Fig. 1D). However, in this case, the steady-state intracellular glucose concentration still remained lower than the one measured with hGluT3 at 5 mM extracellular glucose. Thus, DmGluT1, which can transport glucose with a low affinity but does not transport galactose, is probably not a close "functional" ortholog of hGluT3.

From these results, to investigate whether expression of a glucose transporter could affect the course of a human disease, it seems more relevant to establish *Drosophila* lines expressing a high glucose affinity transporter. For this purpose, we generated flies carrying an insertion of hGluT3 and by crossing them to flies bearing the pan-neuronal *Elav*-Gal4 driver, we could induce hGluT3 expression in control or HQ93 neurons. To validate that our construct was expressed in *Drosophila*, we detected specifically the hGluT3 transcript by RT-PCR in neurons (Fig. 1E).

### Glucose transporter overexpression improves survival of mHtt flies in neurons

**Fig 2:**
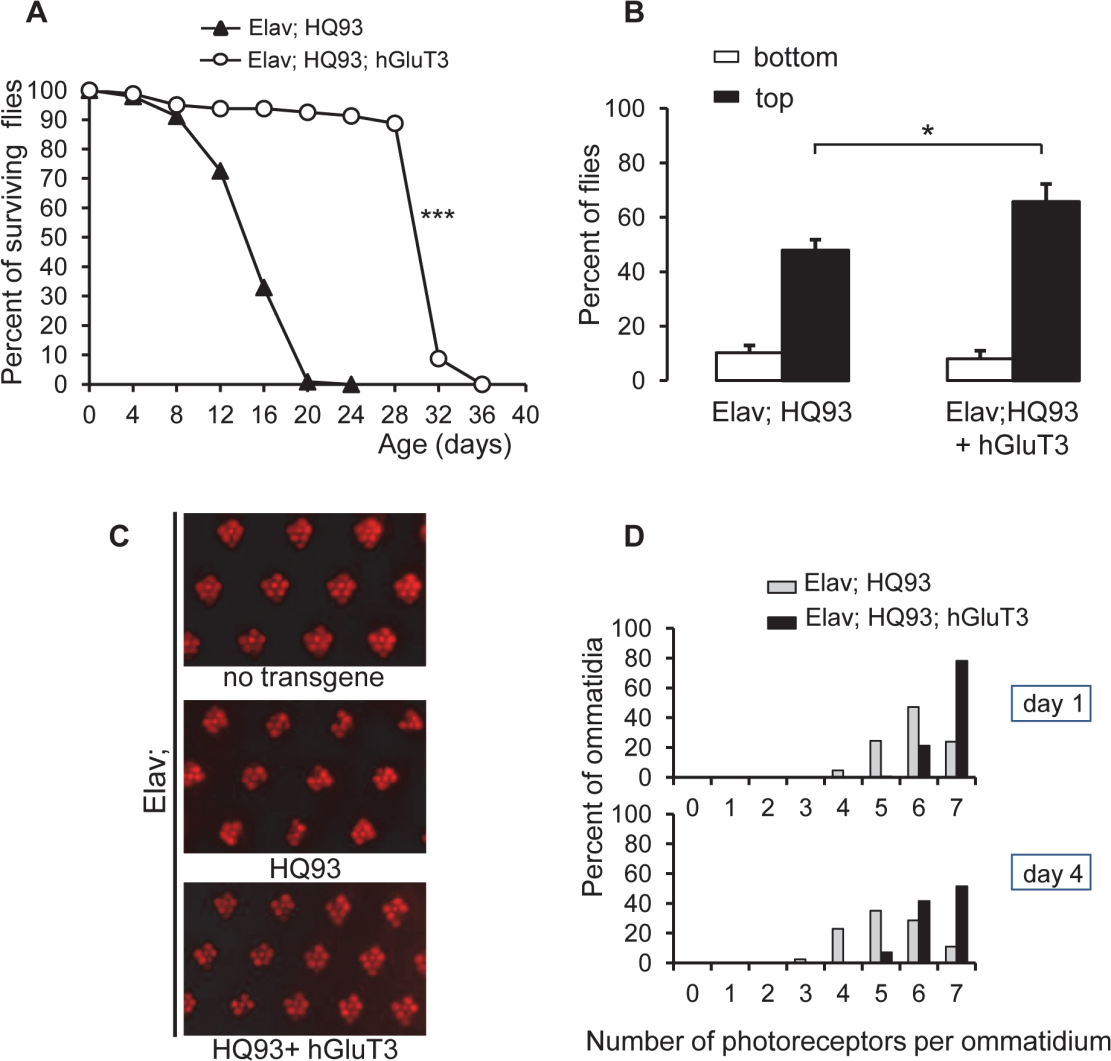
Overexpression of hGluT3 in neurons with HQ93 increases survival, rescues locomotor performance and neurodegeneration. (**A**): Typical survival curve of flies expressing the neuronal driver *Elav-*Gal4 and *HQ93* (filled triangles) or *HQ93* and *hGluT3* (open circles) with n = 237 and 80 flies respectively. The log-rank test indicates that the two survival curves were very different (***; p<0.0001). (**B**): Locomotor performance evaluated by negative geotaxis test on 12 day-old flies expressing the indicated transgenes under the neuronal *Elav-*Gal4 driver. Open columns indicate the percentages of flies remaining at the bottom of the column; filled columns indicate the percentages of flies climbing to the top. Results were the means + SEM of the percentages obtained from a representative experiment (n = 55; 54 flies respectively for each genotype). Comparisons were performed by Student’s t-test (*, p = 0.0239). (**C**): Representative deep pseudopupil photomicrographs of eyes from 4-day old flies expressing no transgene (control), *HQ93* alone, or *HQ93* and *hGluT3* under the control of *Elav-*Gal4. (**D**): Photoreceptor frequency distributions in 1- or 4-day old flies expressing *HQ93* alone, or *HQ93* and *hGluT3*. Statistical significance testing the median value of photoreceptor number per ommatidium was determined by Mann-Whitney test (one-tailed) (p<0.001).

As previously reported, expression of mHtt in *Drosophila* neuronal cells reduces fly longevity [48]. Adults expressing HQ93 displayed a lifespan mean occurring at 17 + 1 days (Fig. 2A) and a maximum lifespan (time to 90% mortality) of 22 + 1 days, whereas lifespan mean of wild-type flies ranges around 66 days (S2 Fig.). When hGluT3 transgene was expressed together with HQ93, the life expectancy mean reached up to 29 + 2 days (Fig. 2A), indicating a 71% increase of lifespan. In the presence of the two transgenes, the maximum survival time was also significantly enhanced since it was 34 + 1 days. We conclude that hGluT3 overexpression is sufficient to significantly improve the survival of the HD flies, by enhancing the mean and maximum lifespans. Expression of hGluT3 did not alter significantly the lifespan mean of control flies (63 days) (S2 Fig.), suggesting that its neuronal overexpression alone has no effect on fly survival. Moreover, HQ20 flies, which express an unexpanded tract of 20 glutamine residues showed no premature death (S3 Fig.) compared to HQ93 flies, in the presence or not of hGluT3. As expected, the survival curve of HQ20 flies was not different from flies expressing no transgene.

### mHtt-induced locomotor impairments are rescued by glucose transporter expression

We utilized a behavioral test, the climbing assay, also named geotaxis negative test which has been extensively used to measure locomotor activity of *Drosophila* [48, 49]. Placed in a column and tapped down, control flies containing the neuronal driver *Elav-*Gal4 alone promptly started to climb along the column and 87% reached the top (S4 Fig.), indicating normal locomotor activity. In contrast, only a reduced fraction of flies expressing HQ93 in neurons (48%) were able to reach the top (Fig. 2B). However, flies expressing HQ93 and the human glucose transporter improved significantly their locomotor performances since 66% flies had reached the top of the column.

### The glucose transporter slows neurodegeneration in HD fly eyes

The fly compound eye, although dispensable for normal development and viability, is a powerful model for analyzing genes contributing to human neurodegenerative diseases [39] and allows to observe neurodegeneration [50]. The *Drosophila* eye is composed of ommatidia with a regular arrangement of seven visible photoreceptor neurons as observed in control by pseudopupil analysis (Fig. 2C) [51]. Expression of HQ93 in neurons led to an obvious loss of one or more photoreceptors leading to a disorganization of ommatidia arrangement. In these flies, the number of ommatidia with seven visible photoreceptors declined from 24% at day 1 to 11% at day 4 after adult emergence (Fig. 2D). This increased loss of photoreceptors was significantly evidenced and showed that neuropathology in the fly is progressive as in the human condition. However, disruption of ommatidia was markedly rescued when the human glucose transporter was co-expressed with HQ93: 78% of ommatidia had 7 photoreceptors one day after adult eclosion and 52% of intact ommatidia were detected at the fourth day of adult life. Overexpression of hGluT3 alone had no effect on the photoreceptors (data not shown).

### Implication of the glycolysis pathway in mHtt *Drosophila* neurons

**Fig 3:**
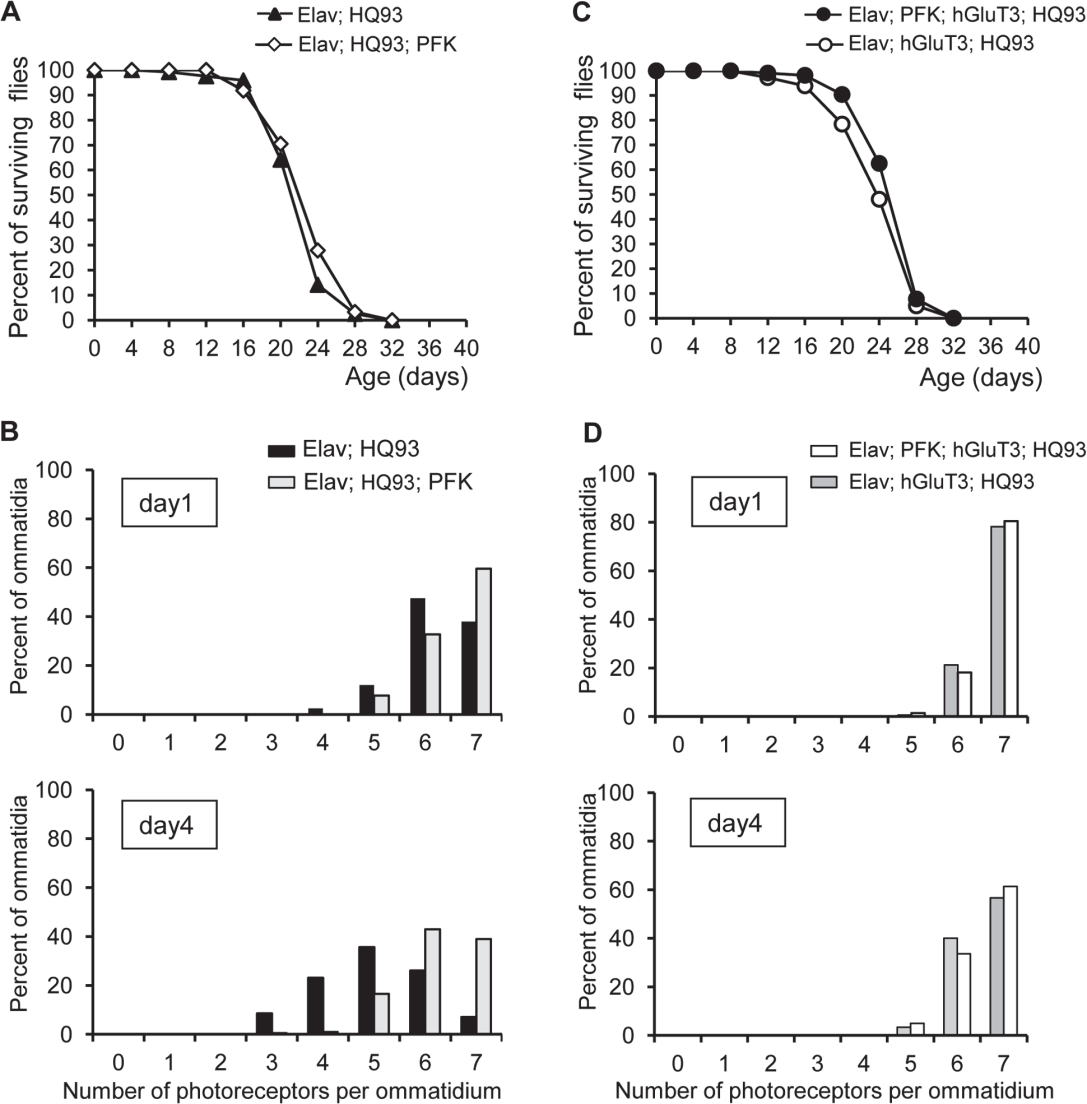
Effect of PFK overexpression on the phenotype of HQ93 flies. (**A**): Lifespans of flies expressing the transgenes *PFK* and *HQ93* (open diamonds) or only *HQ93* (filled triangles) were not different as tested by log-rank test. In this representative experiment, 120 and 61 flies respectively were used. (**B**): Photoreceptor frequency distributions in 1- or 4-day old fly ommatidia expressing *HQ93* alone (black bars), or *PFK* and *HQ93* (grey bars) under the control of *Elav*-Gal4. Statistical significances on median values of photoreceptor numbers per ommatidium were determined by one-tailed Mann-Whitney test (at day 1, p = 0.0033.; at day 4, p< 0.0001). **(C)**: The lifespan of flies expressing *HQ93* together with *PFK* and *hGluT3* (filled circles) was not statistically different from that of flies expressing *HQ93* and *hGluT3* (open circles); in the experiment, 219 and 181 flies were used. (**D**): Photoreceptor frequency distributions in 1- or 4-day old fly ommatidia expressing *HQ93* together with *PFK* and *hGluT3* (white bars), or *HQ93* and *hGluT3* (grey bars) under the control of *Elav*-Gal4. The one-tailed Mann-Whitney test indicates no statistical significances between the two fly lines neither at day 1 nor at day 4.

To investigate the role of glycolysis, we analyzed whether the up-regulation of the glycolytic flux was beneficial in the *Drosophila* HQ93 neurons by overexpressing PFK which catalyses a rate-limiting step between fructose-6-phosphate and fructose-1, 6-biphosphate. For this, we used transgenic flies bearing *Drosophila* PFK [52]. After crossing them with HQ93 flies and *Elav*-Gal4 as driver, we analyzed lifespan and eye neurodegeneration. The Fig. 3A shows that overexpression of this enzyme in neurons did not change the survival of HQ93 flies. However, statistical analysis of the pseudopupil data (Fig. 3B) showed that PFK overexpression prevented neurodegeneration at day 1 and day 4. Since this result could be interpreted as an inefficient import of glucose in brain *sensu stricto* in comparison with the photoreceptors, we analyzed the impact of hGluT3 and PFK co-expression on HD toxicity in the brain and eyes. We showed that PFK and hGluT3 co-expression had no additional effect on survival compared to HD flies expressing hGluT3 alone (Fig. 3C). Similarly, the rescue of photoreceptor degeneration was not enhanced when hGluT3 and HQ93 were co-expressed (Fig. 3D). All these results allow to conclude that overexpression of PFK has no sufficient impact in the brain to rescue longevity of HQ93 flies but was able to delay neurodegeneration in photoreceptors.

### Implication of the pentose-phosphate pathway (PPP) in mHtt *Drosophila* neurons

**Fig 4:**
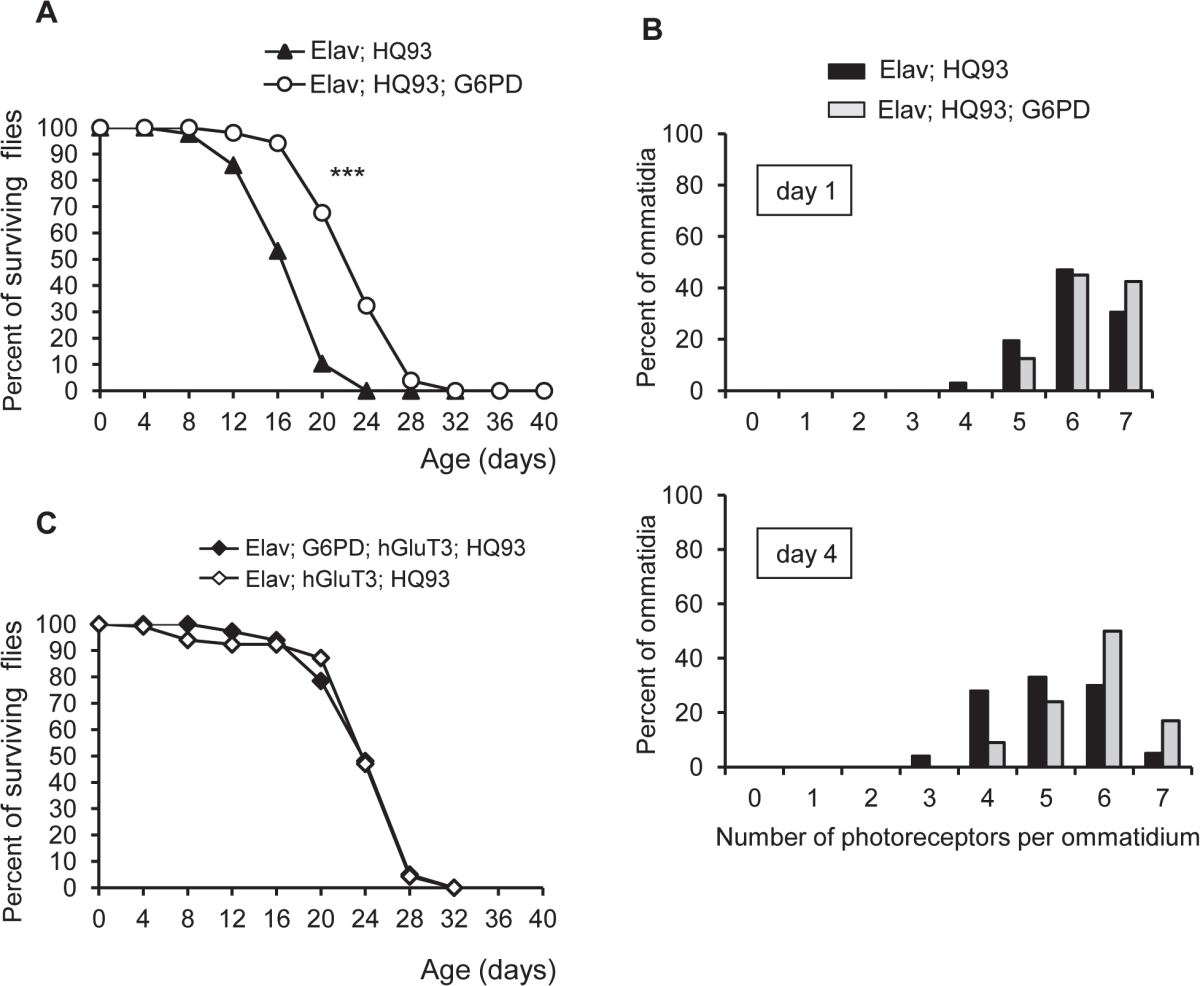
Effect of overexpression of G6PD on the phenotype of HQ93 flies. (**A**): Lifespan of flies carrying two neuronal transgenes *G6PD* and *HQ93* (open circles) was extended in comparison with flies carrying only *HQ93* (filled triangles) with *Elav-*Gal4, n = 102 and 126 flies respectively. Survival curves were highly significantly different by log-rank test (***, p<0.0001). (**B**): Photoreceptor frequency distributions in 1- or 4-day old flies expressing *HQ93* alone (black bars), or *G6PD* and *HQ93* (grey bars). The median value of photoreceptor number per ommatidium between the two lines was statistically significant at the 1^st^ and 4^th^ day after adult emergence (Mann-Whitney test; at day 1, p = 0.0104; at day 4, p< 0.0003). (**C**): Lifespan of *HQ93* flies carrying *hGluT3* and *G6PD* (filled diamonds) was not different from the lifespan of *HQ93* flies carrying only *hGluT3* (open diamonds) with *Elav-*Gal4, n = 117 and 181 flies respectively.

G6PD activity, by counteracting oxidative stress, can protect neuronal cells [15, 53]. We hypothesized that overexpression of G6PD would extend the lifespan of HQ93 flies and enhance their resistance to oxidative stress by its ability to produce NADPH. To perform these experiments, we used a *Drosophila* transgenic line previously characterized, exhibiting a high enzyme activity in the brain and an increase of NADPH content; this line also presented an extension of lifespan and an enhanced resistance to oxidative stress generators as hyperoxia and paraquat treatments [54, 55]. As shown in Fig. 4A, increased expression of G6PD in HD flies was significantly associated with an extension of lifespan: this increase was up to 33% in comparison with flies expressing only the HQ93 transgene. The mHtt-induced neurodegeneration in eyes was significantly rescued by the overexpression of G6PD as seen in Fig. 4B; flies expressing both HQ93 and G6PD have more intact photoreceptor cells (17%) at day 4 after adult eclosion than flies expressing HQ93 alone (5%). To investigate the effects of G6PD on HD fly survival in the presence of hGluT3, we overexpressed HQ93 and G6PD together with hGluT3. As shown in the Fig. 4C, lethality of these flies was not statistically different from that of HD flies expressing hGluT3 alone. This suggests that the co-expression of hGluT3 and G6PD has no cumulative effect on survival rate.

**Fig 5:**
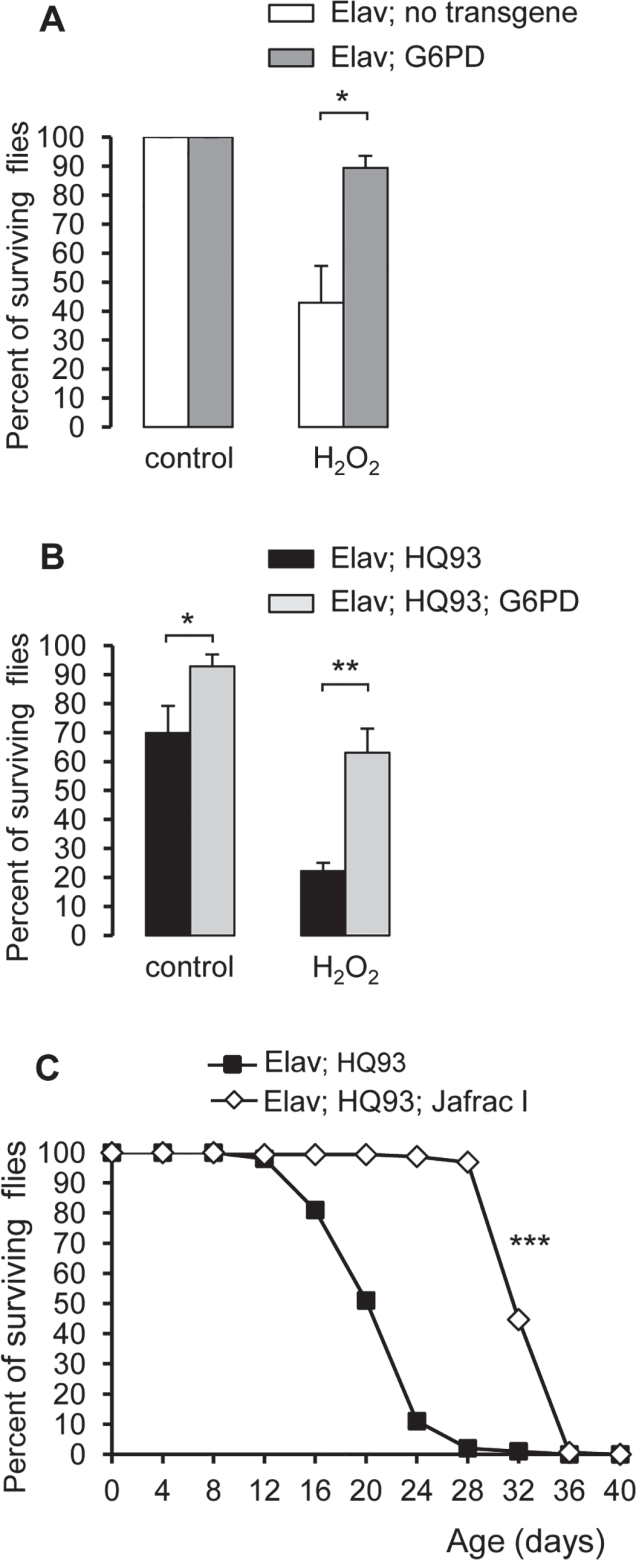
G6PD and JafracI ameliorate oxidative stress tolerance of HD flies. (**A**): Representative survival rate of 12 day-old flies expressing no transgene (white bar) or *G6PD* (grey bar) after 48 hr exposure to 2% sucrose or to 1.5% H_2_O_2_ in 2% sucrose. Numbers of flies included in this assay were respectively: 40; 19; 99; 74. Results represented the means + SEM of the percentages obtained from a representative experiment. The Mann-Whitney test indicates a significant difference between the two genotypes for H_2_O_2_-exposed flies (*, p = 0.016). (**B**): Representative survival rate of 12 day-old flies expressing *HQ93* (black bar), or co-expressing *HQ93* and *G6PD* (grey bar) after 48 hr exposure to 2% sucrose or to 1.5% H_2_O_2_ in 2% sucrose. Numbers of flies included in this assay were 36; 56; 93; 85 respectively. Results represented the means + SEM of the percentages obtained from a representative experiment. The Mann-Whitney test indicates a significant difference between the two genotypes for non-treated flies (*, p = 0.041) and for H_2_O_2_-exposed flies (**, p = 0.006). (**C**): The survival curve of flies expressing *HQ93* as control (squares) or *HQ93* and *Jafrac I* (diamonds) under the neuronal driver *Elav-*Gal4, with n = 100 and 159 flies respectively. The log-rank test indicates that the two survival curves were very different (***, p<0.0001).

Next, we tested resistance of HQ93 flies in the presence or not of G6PD to an oxidizing agent, hydrogen peroxide (H_2_O_2_). At first, we verified that after 48hr exposure, flies with G6PD-expressing neurons were more tolerant to H_2_O_2_ than wild-type flies (Fig. 5A). Secondly, in the presence of HQ93 alone, exposure of 12 day-old flies to H_2_O_2_ drastically reduced fly survival from 70 to 22% (Fig. 5B). Flies with neuronal HQ93 and G6PD transgenes presented a significantly enhanced resistance to oxidant compared to flies carrying only HQ93: 63% of flies with the two transgenes survived in the presence of H_2_O_2_ whereas 22% of HQ93 flies remained alive (Fig. 5B). The amplitude of the decreased lifespan was only 30% with G6PD instead of 48% without G6PD. This result suggests that G6PD was able to rescue fly survival by exerting its anti-oxidative activity even in the presence of mHtt. By contrast, we observed that 6 day-old HQ93 flies were insensitive to oxidative stress generated by oxidative treatment (S5 Fig.), but it is noteworthy that these flies did not yet present pathological symptoms. Further, to determine whether the antioxidant capacity of G6PD in HQ93 neurons was responsible for its positive action, we analyzed the effects of NADPH-dependent peroxidases on fly survival. Peroxiredoxins and thioredoxins have been identified by their ability to reduce oxidant activities in conjunction with thiol-reducing systems using NADPH pool as electron donor [56, 57, 58]. The neuronal expression of *Jafrac1*, a *Drosophila* homologue of the human peroxiredoxin 2 (Fig. 5C) or the *Drosophila* thioredoxin *deadhead* (S6 Fig.) significantly extended the fly lifespan in the presence of HQ93. Thus, G6PD expression and, consequently, activation of the PPP, in addition to their anabolic function, were protective by providing antioxidative supply for mHtt-expressing neurons which are particularly vulnerable to oxidative stress.

### The glucose transporter expression rescues mitochondrial dysfunction

**Fig 6:**
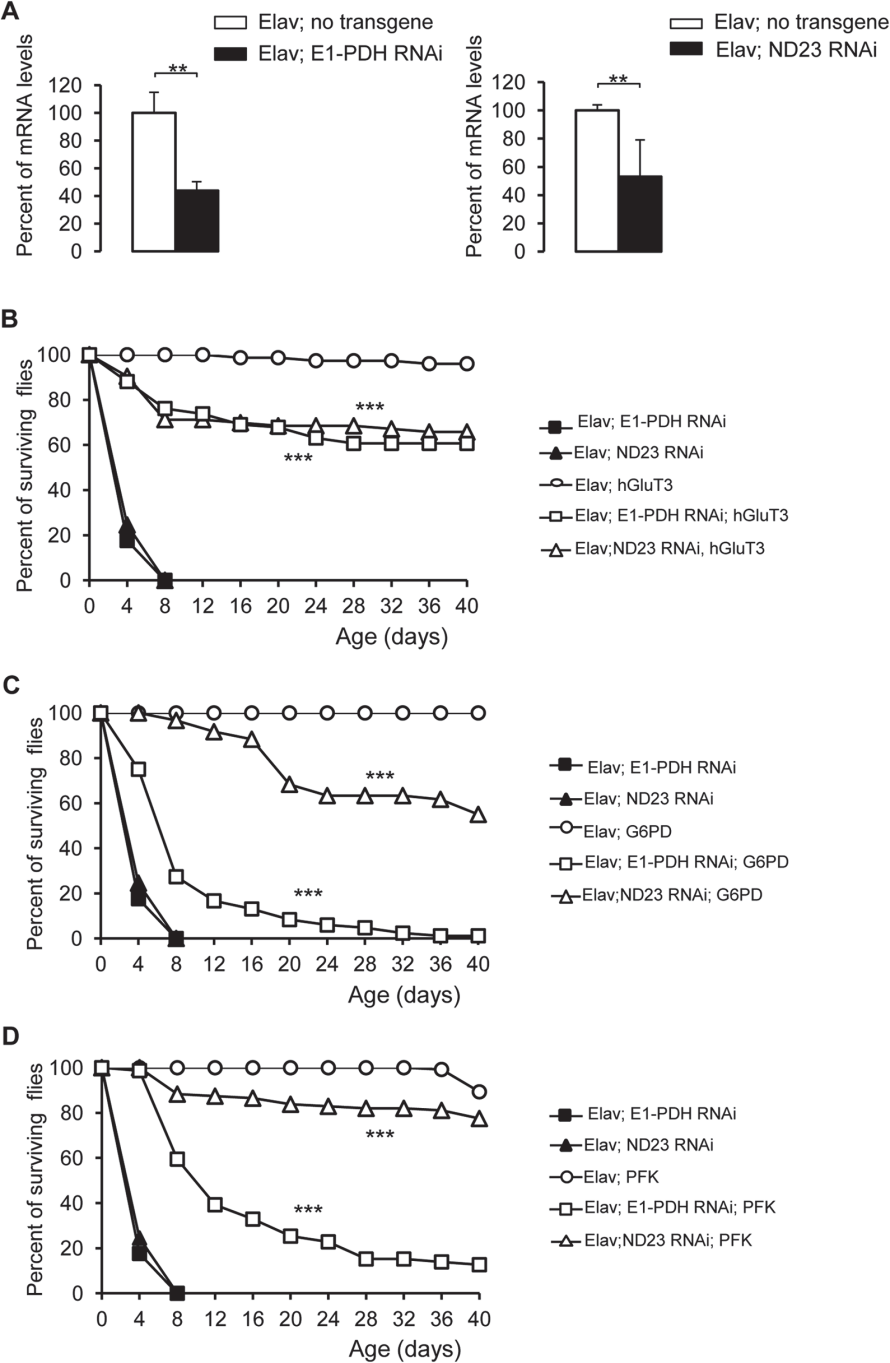
Survivals of flies overexpressing hGluT3 or G6PD or PFK with mitochondrial dysfunctions in neurons. (**A**): RT-qPCR assays for *E1-PDH* and *ND23* transcripts showing reduced levels of each transcript in flies expressing the RNAi under the *Elav*-Gal4 driver. Transcripts levels (filled bars) were expressed in percent relative to controls (no transgene; open bars). They represent the means + SEM of at least 3 separate experiments prepared from heads at the first day of adult age. The one-tailed Mann-Whitney test indicates a difference in mRNA levels between the RNAi-induced and control genotypes (**, p = 0.0076 for *E1-PDH*; **, p = 0.0083 for *ND23*). (**B, C, D**): Survival curves of flies expressing each mitochondria-targeting RNAi alone or together with either *hGluT3*, *G6PD* or *PFK* in the respective figure under the control of *Elav-*Gal4. Only the first fourty days of the lifespan were presented. The survival curves of flies expressing only the RNAis specifically-targeted to *E1-PDH* (filled squares; n = 51) and *ND23* subunits (filled triangles; n = 53) were in common for the figures B, C and D. The survival for control flies (*Elav; hGluT3* or *Elav; G6PD* or *Elav; PFK*) was indicated by open circles in each corresponding graph (n = 75; 81; 131, respectively). In each figure, the log-rank test indicates a *p*-value <0.0001 (***) between the survival curves of the flies overexpressing *hGluT3* or *G6PD* or *PFK* and the RNAi and the survival curves of the flies expressing each RNAi only. (**B**): Survival curves of flies expressing the RNAis or co-expressing each respective RNAi and *hGluT3* (open symbols; n = 84 and 73) under *Elav-*Gal4. (**C**): Survival curve of flies expressing the RNAis or co-expressing each respective RNAi and *G6PD* (open symbols; n = 84 and 60 respectively) under *Elav-*Gal4. (**D**): Survival curve of flies expressing the RNAis or co-expressing each respective RNAi and *PFK* (open symbols; n = 79 and 111 respectively) under *Elav-*Gal4.

An increasing number of studies have shown that mutant Htt action results in mitochondrial dysfunction [59, 60]. We tested whether or not increasing glucose metabolism could prevent effects of mitochondrial dysfunction. Therefore, we examined the impact of hGluT3 neuronal overexpression on fly lifespan after genetic inactivation of two key genes for mitochondrial activity: the pyruvate dehydrogenase complex and the mitochondrial respiratory system. In mitochondrial matrix, the pyruvate dehydrogenase complex (PDH) catalyses the conversion of pyruvate to acetyl-coA and constitutes the first step of the TCA cycle. The mitochondrial respiratory complex I contains evolutionary conserved NADH ubiquinone oxidoreductase complex components; it produces significant amounts of ROS and its dysfunction triggers oxidative impairment as observed in several neuropathological diseases [17, 61, 62]. Firstly, we verified by RT-qPCR analysis that RNA interference (RNAi) expressions in neurons have efficiently reduced the expression of their respective targets: the alpha-subunit of the acetyl-transferring component of the PDH complex (E1-PDH) and the 23kD subunit (ND23) in the complex I (Fig. 6A). The Fig. 6B shows that knockdown of these both genes in neurons led to a dramatic reduction of the lifespan with a expectancy mean of 5 and 4 days respectively; this shows the key role of mitochondria in neuronal functions. However, when the hGluT3 transgene was expressed together with each RNAi, life expectancies were very significantly rescued since the means reached up 56 days with the E1-PDH RNAi and 59 days in the case of the ND23 RNAi (Fig. 6B). These results indicate that hGluT3 overexpression was sufficient to rescue the survival of the flies presenting mitochondrial defects. Then, to test the respective roles of the glycolysis or PPP to counteract mitochondrial dysfunction, we overexpressed the key enzymes G6PD (Fig. 6C) or PFK (Fig. 6D) in neurons in the presence of each RNAi. Both enzymes rescued the survival of the E1-PDH or ND23 RNAi expressed in neurons. This suggests that an increase in glycolysis and/or in PPP was required to maintain cell survival in stress conditions following impairment of mitochondrial functions.

## Discussion

In the present work, using a genetic approach, we provided the first evidence that increasing metabolism of glucose sustained by overexpression of the glucose transporter hGluT3 ensures neuronal maintenance and survival in HD pathology. Previously, we have showed that DmGluT1, a predicted *Drosophila* sugar transporter ameliorates the survival of HQ93 flies when it was expressed in glial cells [45]. We also found that DmGluT1 expressed in neurons confers protection against mHtt (data not shown), and this result has been recently confirmed [28]. However, although having striking amino acid homology (44–49%) with the human classical glucose transporters (GluT1–4), we showed here that DmGluT1 has low affinity for glucose and galactose contrary to hGluT3. Moreover, it was transcribed at a very low level in wild-type fly heads (data not shown). On the basis of these data, we concluded that this isoform is probably not implicated in *Drosophila* neuronal glucose import and that DmGluT1 is likely not the neuronal “functional” ortholog of the hGluT3, although this role has been suggested in the report of Vittori *et al*. [28]. However, we cannot exclude that DmGluT1 may transport an undetermined or modified sugar. Thus, consequently, we focused our study on hGluT3 overexpression in *Drosophila* neurons. We showed that expression of this glucose transporter in *Drosophila* neurons is sufficient to suppress most of the neurological mHtt-induced phenotypes by strikingly improving fly survival, restoring locomotor activity and rescuing neurodegeneration. Our data confirm that HD progression could be affected by hGluT3 gene expression level and its glucose uptake activity in neurons.

In physiological conditions, after its import, intracellular glucose is phosphorylated and is thus ready to enter metabolic pathways, mainly glycolysis or PPP. But in HD, functions of genes related to carbohydrate metabolism and acting downstream glucose transporters are altered [63, 64, 65]. Diverse mechanisms have been proposed which could underlie the hypometabolism observed: decrease of glycolysis activity [66, 67], deregulation of mitochondrial metabolism [68] including high ROS production [69] or ATP depletion [26].

To test the effect of the increasing glycolytic flux in mHtt transgenic flies, we used the PFK enzyme which catalyses an irreversible step of the glycolysis and is known as a regulator of this pathway. We found that overexpression of PFK in neurons had an overall modest impact on HD progression. This is not due to a low import of glucose in fly neurons since increasing glucose entry by hGluT3 did not reveal a beneficial effect of PFK. However, the absence of an efficient glycolytic effect on fly survival could be explained by the continuous degradation of the PFK or by a functional blockage at the PFK step as described in mammals [70]. Moreover, glycolysis up-regulation might have adverse effects since stabilization of the neuron-specific isoform of PFK by an excitotoxic stimulus increased neuronal glycolysis but thereafter led to oxidative damage and cell death [71]. It has been also demonstrated that GAPDH, a key enzyme in the glycolytic pathway downstream of the PFK step, can mediate neurotoxicity by binding to the polyglutamine stretch of the mutated huntingtin [72, 73]. Inhibitors of glycolysis were found to suppress cell death in a cell culture model expressing poly-Q [74], suggesting that enhancement of the glycolysis may exacerbate the toxic action of mHtt in neurons. In conclusion, activation of glycolysis does not appear as an efficient pathway to compensate HD-induced energy deficits.

Neurons are the most sensitive cells of the brain to oxidative damage [75]. The PPP largely contributes to neuronal protection against oxidative injury by the G6PD activity which reduces NADP to NADPH, a necessary cofactor for regeneration of glutathione or thioredoxin in the reduced form. Several studies have shown that the PPP is up-regulated when the brain is subjected to traumatic injury or abnormal oxidative stress [76] and appears to play a critical role during neurological diseases [77]. It is well documented that HD pathological conditions augment production of ROS in patients and in HD models [78]. Here, we have showed that G6PD overexpression in neurons was beneficial for HQ93 fly survival and produced eye neuroprotection. G6PD and hGluT3 overexpressions seem to have no cumulative effects on the lifespan of HD flies, further indicating that these two genes are involved in the same metabolic pathway. We also showed that flies expressing G6PD in neurons in the presence of HQ93 presented a greater resistance to H_2_O_2_-induced oxidative stress than flies expressing only HQ93, indicating that the rescuing effect of G6PD was probably mediated by increased NADPH production. It has been reported that polyglutamine toxicity in *Drosophila* eyes was reduced by increasing NADPH level through elevated G6PD activity [55]. Furthermore, we showed that ROS detoxifying enzymes belonging to the *Drosophila* redox buffer system [57] such as thioredoxins and peroxiredoxins, conferred protection in neurons expressing HQ93. This result was in agreement with the neuroprotective effect of the mouse glutathione peroxidase in HD flies [79]. We thus propose that neurons overwhelmed by the pleiotropic action of mHtt, became more and more vulnerable to mHtt-induced ROS overload, and that the up-regulation of the PPP is a necessary protective antioxidant strategy against HD by reducing the amounts of ROS.

Mitochondrial abnormalities has long been proposed to underlie neuronal loss in HD pathology and during recent years, numerous studies focused on the role of mitochondrial dysfunction in the disease [68, 80]. To investigate the effects of glucose metabolism on impaired mitochondrial function, RNAis were used to reduce the respective activities of the TCA cycle and complex I by specifically silencing the E1 component of the PDH complex and the 23kD subunit of the NADH ubiquinone oxidoreductase complex. Our data showed that the knockdown of these two targets reduced drastically fly survival and that overexpressions of either hGluT3, G6PD or PFK ameliorated lifespan. It was proposed that mitochondria can adjust cellular bioenergetic performance and alleviate stress by inducing metabolic readjustments through a feedback mechanism, the mitochondrial retrograde signal. It has been firstly characterized in yeast [81] and represents coordinated cellular responses to changes in the functional state of mitochondria to promote cell survival. Recent studies in *Drosophila* or in a *Drosophila* cell line also point to a retrograde response to support cell survival and activity during mitochondrial stress [82, 83]. Our results show that overexpression of genes involved in glucose metabolism may compensate for mitochondria-induced alterations. Nevertheless, further studies are required to determine whether or not such compensatory mechanisms occur in the HD context and particularly, prior to the onset of symptoms.

In conclusion, we show that the unique feature of increased glucose neuronal import by increasing neuronal transporter expression has a striking beneficial impact on HD pathology to maintain neuronal activities. The induction of PPP is able to delay HD progression likely by providing efficient neuroprotection against ROS. In contrast, the enhancement of glycolysis is probably not a preferential way since glycolysis might promote neurotoxicity by compromising the efficacy of the antioxidant system. These observations could contribute to find future therapeutic strategies implying the neuronal glucose metabolism.

## Material and Methods

### *Drosophila* Stocks

Flies were raised at 25°C on standard cornmeal agar diet. The *UAS-Htt exon 1-Q93* flies, hereafter named HQ93 flies, and *UAS-Htt exon1-Q20* flies (named HQ20 flies) were provided by L. Marsh and had been described in [43]. The transgenic overexpression strains: *UAS-PFK* (line #4) were kindly provided by C.S. Thummel; the *UAS-G6PD* (line #5f) flies were supplied by W.C. Orr, the *UAS-Jafrac I* flies by W-J. Lee and the *UAS-deadhead* flies by T. Aigaki. The neuronal driver *Elav-*Gal4 (line c155) was obtained from the Bloomington *Drosophila* Stock Center (Bloomington, Indiana). The RNAi lines *UAS-ND23-IR* (#110797) and *UAS-E1-PDH-IR* (#40410) were purchased from VDRC (Vienna, Austria). Accordingly to the genetic background of the different lines, we used as control either the *yw* or the *w* ^*1118*^ (BL5905) lines (S1 Table). To proceed to neuron-specific expression, flies carrying one or two UAS constructs were crossed to flies transgenic for the pan-neuronal *Elav-*Gal4 driver. Female F1 progeny carried both UAS and Gal4 were used for subsequent analyses.

### Lifespan experiments

Newly eclosed adult female flies of the appropriate genotypes were collected within 24 hrs of emergence in vials at a density of about 20 flies per vial and maintained at 25°C. Flies were transferred every 2–3 days to fresh food, and the number of dead flies counted each day. Two to eight experiments were conducted in the same conditions and representative survival curves were shown. Survival curves were generated and statistical significance was tested by using log-rank statistics software (GraphPad Prism).

### Locomotion assay

Locomotor performance was tested by the negative geotaxis test [48]. Briefly, female flies were anesthetized with CO_2_ and placed in a plastic column. After 30 min recovery, flies were gently tapped to the bottom of the column, then allowed to climb for 30 sec. The test was repeated 3 times for each batch of flies at 1 min intervals. For each experiment, the percentages of flies that reached the top of the column and that remained at the bottom were separately calculated. Statistical significance was assessed using the Student’s t-test (GraphPad Prism).

### Pseudopupil analysis

One eye of adult female head was dipped in vaselin grease covering a microscope slide. Eyes were observed with a Leica TCS SP2 microscope using a x60 objective, and photographed with a CoolSnaps HQ Photometrics camera. The visualization of the trapezoidal arrangement of photoreceptor cells in the ommatidia was performed with Image J. At least 10 flies were examined per genotype and the numbers of visible rhabdomeres were scored for 20 ommatidia per fly. Comparisons between median value of photoreceptor number per ommatidium in each line were performed using one-tailed Mann-Whitney’s test (GraphPad Prism).

### *Drosophila* hGluT3 generation

The cDNA of hGluT3 in pCMV-Sport6 plasmid (IMAGE clone: 4396508, ImaGenes, Berlin, Germany) was PCR amplified with the following primers: forward: 5’ TTTGGATCCTTCCTGAGGACGTG and reverse: 5’ TATCCTCGAGGGATACTCTAGAG, then digested with BamH1 and NotI restriction enzymes, respectively. The purified fragment (3.3 kb) was inserted into the BglII and NotI restriction sites of the pUAST plasmid. The selected clone was verified by DNA sequencing. Germ-line transformation was performed by BestGene (BestGene Inc., USA) in a *yw* background.

### DmGluT1 and DsRed-tagged DmGluT1 constructions

For non tagged-DmGluT1 contruction, the pUAS-DmGluT1 plasmid [45] was used to excise a 2.5 kb DmGluT1 sequence using EcoRI and XhoI restriction enzymes. The purified DmGluT1 fragment was inserted in the EcoRI and XhoI sites of the pcDNA3 plasmid (Life Technologies, USA), then ligated. The ligation boundaries were verified by sequencing.

The pDsRed-Monomer-N1 (Clontech Laboratories) plasmid was used to construct fusion of the DmGluT1 coding sequence to the N-terminus of the DsRed sequence. The pUAS-DmGluT1 plasmid was amplified with primers containing XhoI and BamHI restriction enzymes respectively, to insertion in the multiple cloning site of the DsRed vector: forward: 5' TTTTCTCGAGGCAACTGGCAACGAAATGGCT and reverse: 5' TTTTGGATCCACATACCTGCCATTGTTGTGC. After digestions and purification, the fragment (1.5 kb) was inserted into the DsRed vector, then the sequence of the construct was verified by sequencing.

### Glucose and galactose intracellular measurements

HEK-293T cells (American Type Culture Collection) were co-transfected at 30% confluence using Lipofectamine 2000 (Life Technologies, USA) and 0.5 μg/ml plasmid DNA coding for the FRET glucose sensor FLII^12^Pglu700μΔ6 [84] and either control (mock or DsRed plasmids), hGluT3, DmGluT1 or DsRed-tagged DmGluT1 vectors. After 24 hours, cells were superfused with a bathing solution containing (in mM) 136 NaCl, 5 KCl, 1.25 CaCl2, 1.25 MgCl2, 2 glucose, 10 HEPES pH 7.4, and imaged at RT with an Olympus FV 1000 laser confocal microsocope. Steady-state intracellular glucose concentrations at 5 or 25 mM extracellular glucose, glucose clearance rate and galactose uptake were estimated as previously described [84, 85].

### hGluT3 immunodetection and Dsred-tagged DmGluT1 detection

For hGluT3 immunocytochemistry, cells were 4% formaldehyde fixed (20 min) 24 hrs after transfection and then permeabilized with 0.2% Triton X-100 for 10 min and treated with 50mM NH_4_ Cl (10 min). Cells were incubated in 1% BSA-PBS for 5 min (x3) to block non-specific interactions. Cells were then incubated with the primary antibody used at a 1:100 dilution (rabbit anti C-term GluT3; Abcam) for 2h at RT. The secondary antibody was Dy549 goat anti-rabbit (Jackson) used at a 1:500 dilution for 1h at RT. Cultures were imaged with an Olympus FV 1000 laser confocal microscope. For DsRed fluorescent detection, cells were 4% formaldehyde fixed (10 min) and confocal image acquisition was performed on a Zeiss LSM780 laser scanning microscope.

### Reverse transcription-PCR

Total RNA was extracted from 10–15 heads of 6-day post-eclosion flies using Trizol reagent (Life Technologies, USA). RNA concentration and purity were measured with a Nanodrop spectrophotometer (NanoDrop 1000, v3.7.1, Thermo Fischer Scientific, USA). The cDNAs were synthesized from 1 μg of mRNA template and oligo-dT primer (Euromedex, France) using ImProm-II Reverse Transcriptase (Promega, France) at 42°C following the manufacturer’s instructions. PCR parameters were for 39 cycles: 30 s at 92°C, 30 s at 61°C and 45 s at 72°C with the following primers: 5’-GGAGTCCAGGGAAGAGAAAGT and 5’-GTATGTGCACTGTCACTTTGC. Actin 5C primers (5′-CGTTTACAGTAGTTTTCACGCC and 5′-CACTTGCGGTGCACAATGGA) amplify a 1180 bp cDNA fragment and a 1773 bp genomic DNA fragment to check for potential genomic DNA contamination. PCR products were separated on 1% agarose gels and visualized by SybrSafe (Life Technologies) staining. Total RNA samples for RT-PCR were independently prepared 3 times.

### Quantitative PCR

Reactions were monitored by using ABI Prism 7500 Fast thermal cycler (Life Technologies, USA) and KAPA SYBR Fast qPCR Master mix (Clinisciences, France) on reverse-transcribed cDNAs as obtained above. Cycling parameters were used: 40× (95°C for 3 sec, 60°C for 30 sec). Primers were designed with AmplifX version 1.7.0 software (http://crn2m.univ-mrs.fr/pub/amplifx-dist; CNRS, Aix-Marseille University) and are listed in the S2 Table. Data were analyzed using the relative quantification method (-2 ΔCt method). The transcript levels were normalized with values obtained after amplification of ribosomal RNA (rp49) as endogenous control. Serial dilutions from 1:1 to 1:625 cDNAs were used for each gene and for the internal control to generate standard curves to check a nearly 100% efficiency. Data represent two averaged replicates of at least three independent experiments, each of which was carried out on separate set of tissue samples. Melting curves were established for each reaction to check that only one specific amplicon was synthesized during the amplification. Excel software (Microsoft) was used to analyze data and to generate representative graphs expressing mean expression levels + SEM. Expression levels were expressed in percent relative to controls, then compared with Mann-Whitney test (GraphPad Prism).

### Hydrogen peroxide resistance

Female adult *Drosophila* were collected within 24 hrs of emergence and transferred into vials with food in groups of 20 flies per vial. Six or twelve days after emergence, *Drosophila* were tested at 25°C for resistance to H_2_O_2_ (Sigma-Aldrich, USA) treatment. Flies were starved for 2 hrs, then placed into vials containing Whatman paper pieces saturated with 1.5% H_2_O_2_ (v:v) in 2% sucrose or with 2% sucrose alone as control. Numbers of dead flies were recorded at 48 hrs after the beginning of the treatment. Two independent trials were performed, and a representative experiment was shown. One-tailed Mann-Whitney tests (GraphPad Prism) were used to determine the significance of the data expressed in percentages of surviving flies.

## Supporting Information

S1 Fig Immunodetection of hGluT3 and the glucose sensor. After co-tranfection with the FRET glucose sensor FLII^12^Pglu700μΔ6 (left panel) and *hGluT3* (central panel), HEK-293 cells showed localization of the two labellings (right panel): *hGluT3* was mainly located to plasma membrane and cytoplasm whereas the sensor was mostly cytoplasmic. Confocal epifluorescence microscopy was performed at × 63. Scale bar is 50 μm.
(TIF)

S2 Fig Overexpression of hGluT3 in control flies has no effect on longevity. Survival curve of flies expressing no transgene (control; circles) or *hGluT3* (triangles) under the neuronal driver *Elav-*Gal4. The log-rank test indicates no significant difference.
(TIF)

S3 Fig Survival of flies expressing an unexpanded polyglutamine tract (HQ20) in neurons. Under the neuronal driver *Elav-*Gal4, 98% flies expressing HQ20 (filled triangles; n = 109) and 98% flies with no transgene (open diamonds; n = 90) were alive at 40 days of age, whereas 100% of *HQ93* flies (white triangles; n = 97) were died. The presence of *hGluT3* ameliorated the survival of *HQ93* flies (open circles; n = 106) and has no effect on *HQ20* flies (filled circles; n = 112).
(TIF)

S4 Fig Locomotor performance in control flies. Negative geotaxis test was assayed on 12 day-old flies expressing no transgene under the neuronal *Elav-*Gal4 driver. Open column indicates the percentages of flies remaining at the bottom of the column; filled column indicates the percentages of flies climbing to the top. Results were the means + SEM of the percentages obtained from a representative experiment (n = 55 flies).
(TIF)

S5 Fig Resistance to H2O2-induced stress was not altered in neurons of 6 day-old flies. (**a**): Representative survival rate of 6 day-old flies expressing no transgene (white bar) or *G6PD* (grey bar) after 48 hr exposure to 2% sucrose or to 1.5% H_2_O_2_ in 2% sucrose. Numbers of flies included in this assay were: 35; 58; 75; 101 respectively. Results represented the means + SEM of the percentages obtained from a representative experiment. The Mann-Whitney test indicates no significant difference. (**b**): Representative survival rate of 6 day-old flies expressing HQ93 (black bar), or co-expressing HQ93 and G6PD (grey bar) after 48 hr exposure to 2% sucrose or to 1.5% H_2_O_2_ in 2% sucrose. Numbers of flies included in this assay were 67; 49; 97; 119 respectively. Results represented the means + SEM of the percentages obtained from a representative experiment. The Mann-Whitney test indicates no significant difference.
(TIF)

S6 Fig Survival of flies overexpressing the *Drosophila* thioredoxin *deadhead* and HQ93 in neurons. Lifespan of flies expressing the two transgenes *dhd* and *HQ93* (open circles) was extended in comparison with control flies (filled triangles) with *Elav-*Gal4, n = 129 and 140 flies respectively. Survival curves were highly significantly different by log-rank test (***, p<0.0001).
(TIF)

S1 Table Control lines according to the genetic background of the *Drosophila* lines used in experiments. (DOC)

S2 Table List of primers used for qPCR. (DOC)
