# Supplementary figures and images for: Enhanced Neuronal Glucose Transporter Expression Reveals Metabolic Choice in a HD Drosophila Model
Source: PLoS One. 2015 Mar 11;10(3):e0118765. doi: 10.1371/journal.pone.0118765 (PMC4356621; doi:10.1371/journal.pone.0118765)

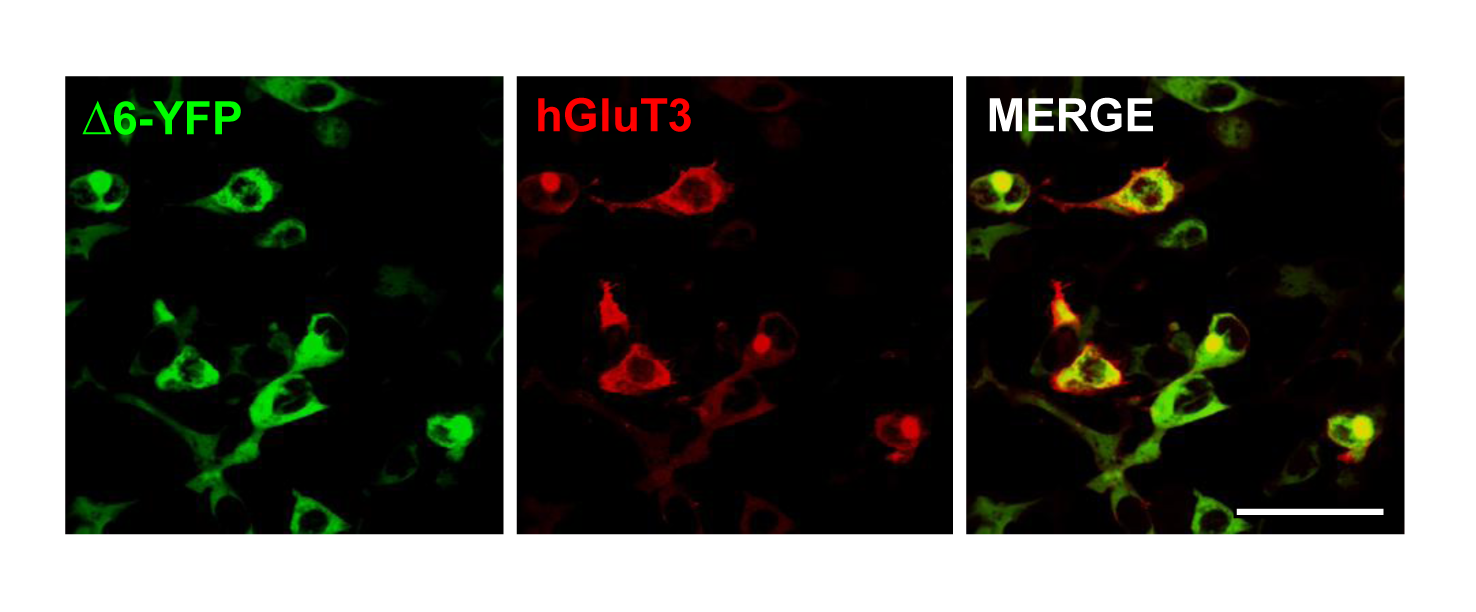

Supplement: S1 Fig — After co-tranfection with the FRET glucose sensor FLII12Pglu700μΔ6 (left panel) and hGluT3 (central panel), HEK-293 cells showed localization of the two labellings (right panel): hGluT3 was mainly located to plasma membrane and cytoplasm whereas the sensor was mostly cytoplasmic. Confocal epifluorescence microscopy was performed at × 63. Scale bar is 50 μm. (TIF) [file pone.0118765.s001.tif]

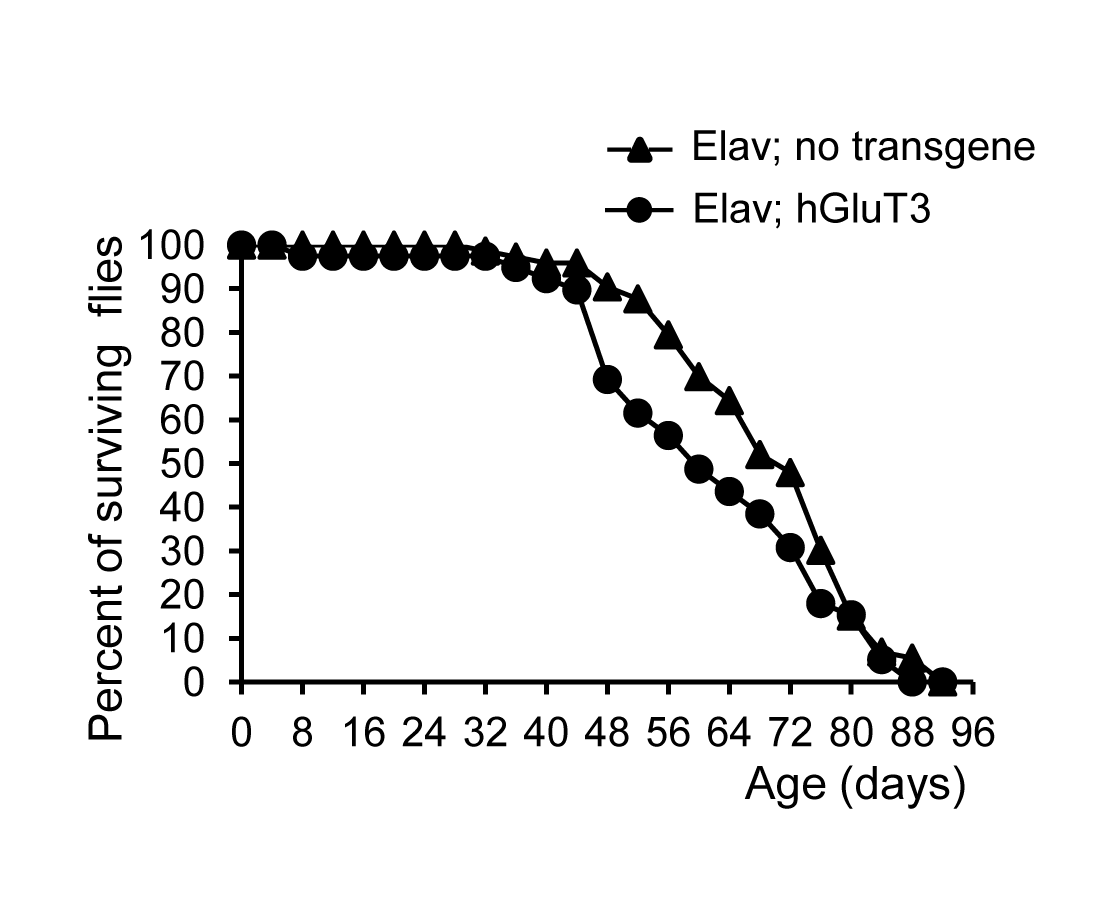

Supplement: S2 Fig — Survival curve of flies expressing no transgene (control; circles) or hGluT3 (triangles) under the neuronal driver Elav-Gal4. The log-rank test indicates no significant difference. (TIF) [file pone.0118765.s002.tif]

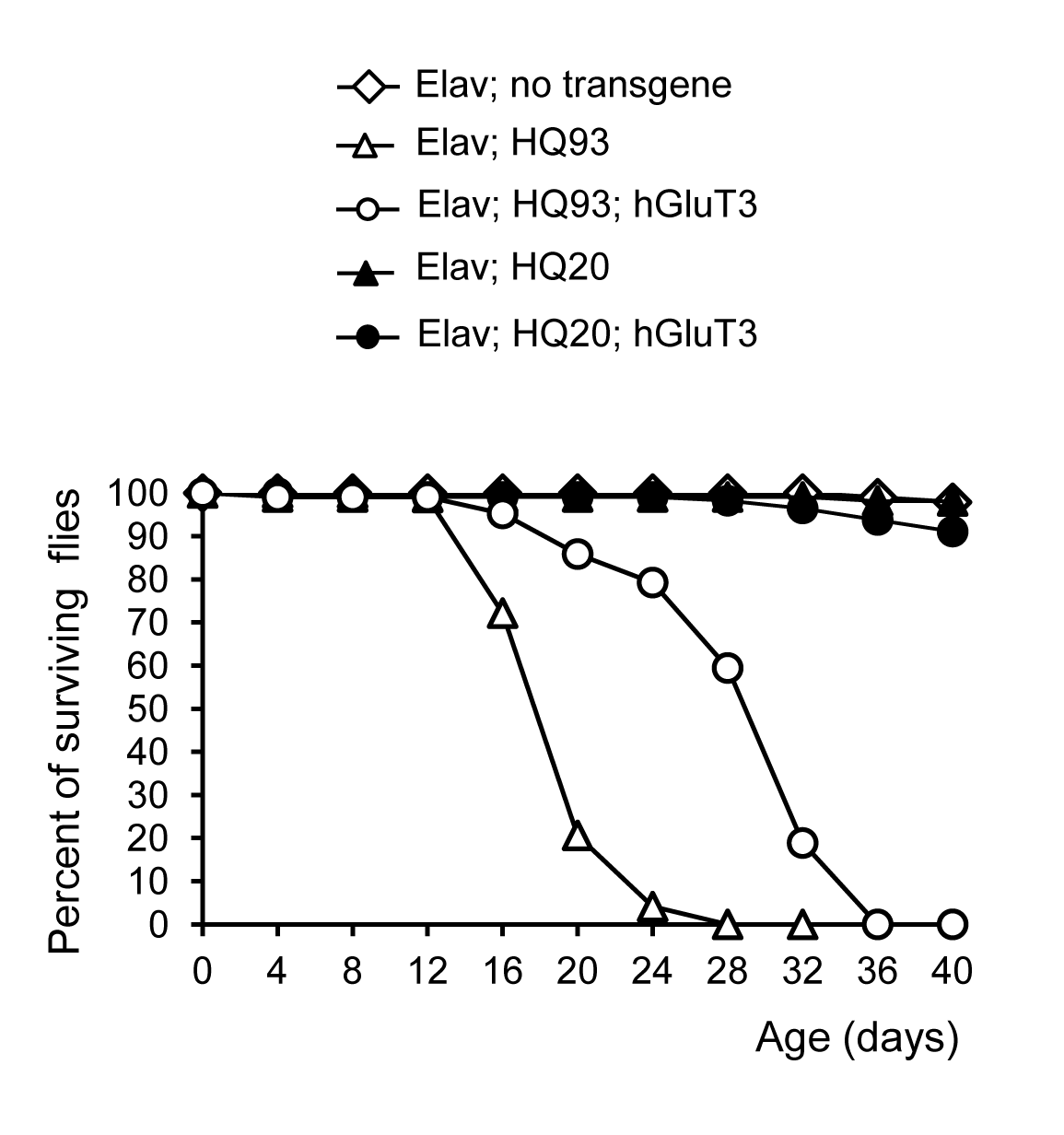

Supplement: S3 Fig — Under the neuronal driver Elav-Gal4, 98% flies expressing HQ20 (filled triangles; n = 109) and 98% flies with no transgene (open diamonds; n = 90) were alive at 40 days of age, whereas 100% of HQ93 flies (white triangles; n = 97) were died. The presence of hGluT3 ameliorated the survival of HQ93 flies (open circles; n = 106) and has no effect on HQ20 flies (filled circles; n = 112). (TIF) [file pone.0118765.s003.tif]

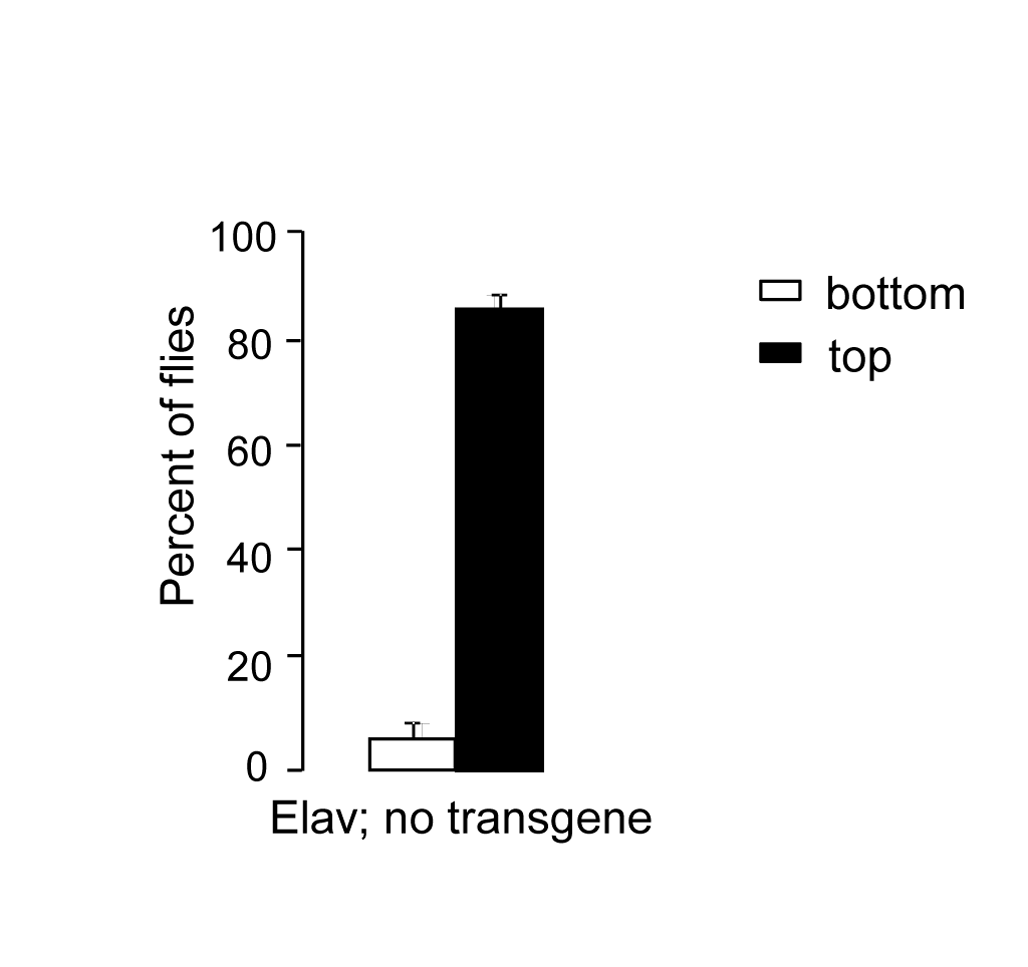

Supplement: S4 Fig — Negative geotaxis test was assayed on 12 day-old flies expressing no transgene under the neuronal Elav-Gal4 driver. Open column indicates the percentages of flies remaining at the bottom of the column; filled column indicates the percentages of flies climbing to the top. Results were the means + SEM of the percentages obtained from a representative experiment (n = 55 flies). (TIF) [file pone.0118765.s004.tif]

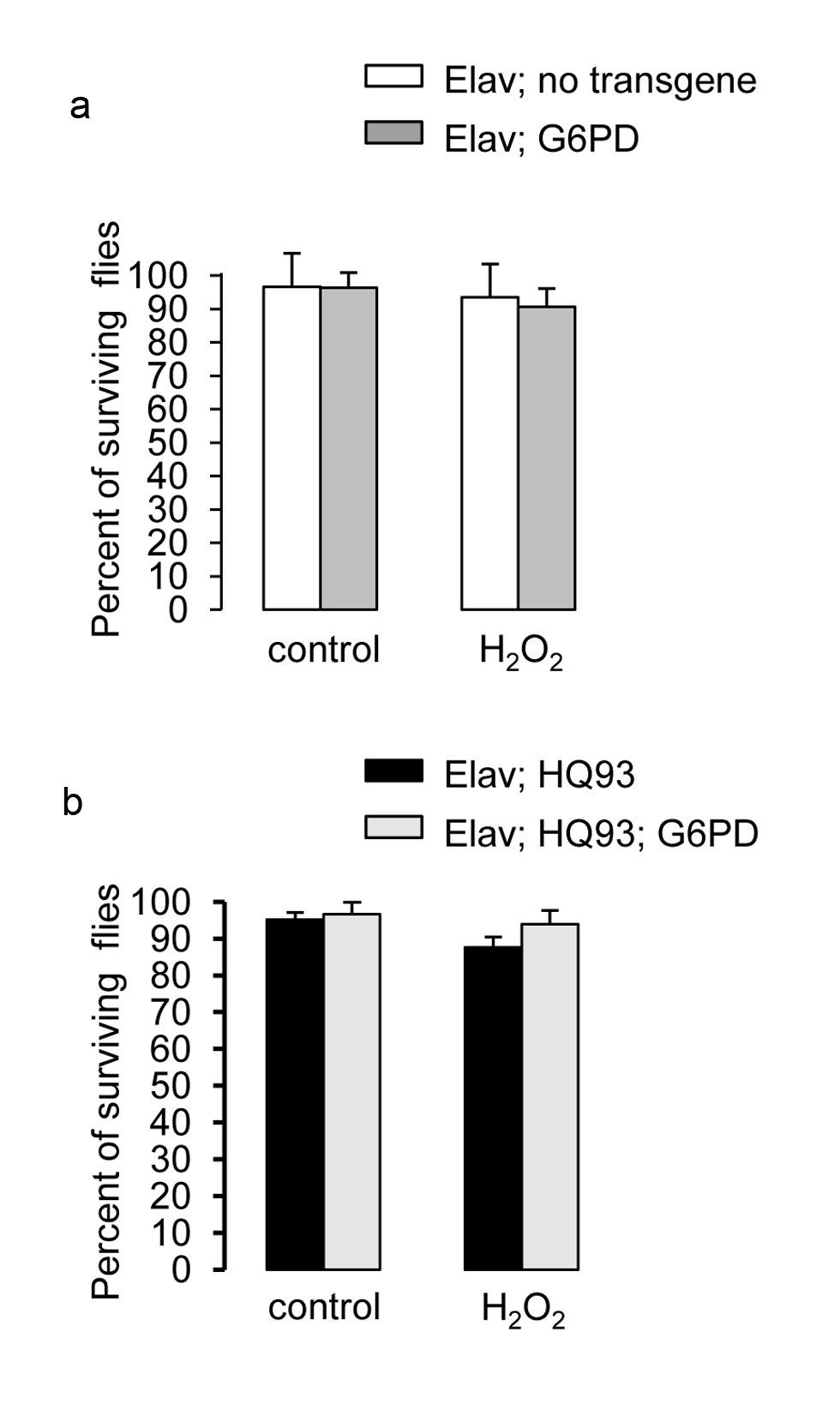

Supplement: S5 Fig — (a): Representative survival rate of 6 day-old flies expressing no transgene (white bar) or G6PD (grey bar) after 48 hr exposure to 2% sucrose or to 1.5% H2O2 in 2% sucrose. Numbers of flies included in this assay were: 35; 58; 75; 101 respectively. Results represented the means + SEM of the percentages obtained from a representative experiment. The Mann-Whitney test indicates no significant difference. (b): Representative survival rate of 6 day-old flies expressing HQ93 (black bar), or co-expressing HQ93 and G6PD (grey bar) after 48 hr exposure to 2% sucrose or to 1.5% H2O2 in 2% sucrose. Numbers of flies included in this assay were 67; 49; 97; 119 respectively. Results represented the means + SEM of the percentages obtained from a representative experiment. The Mann-Whitney test indicates no significant difference. (TIF) [file pone.0118765.s005.tif]

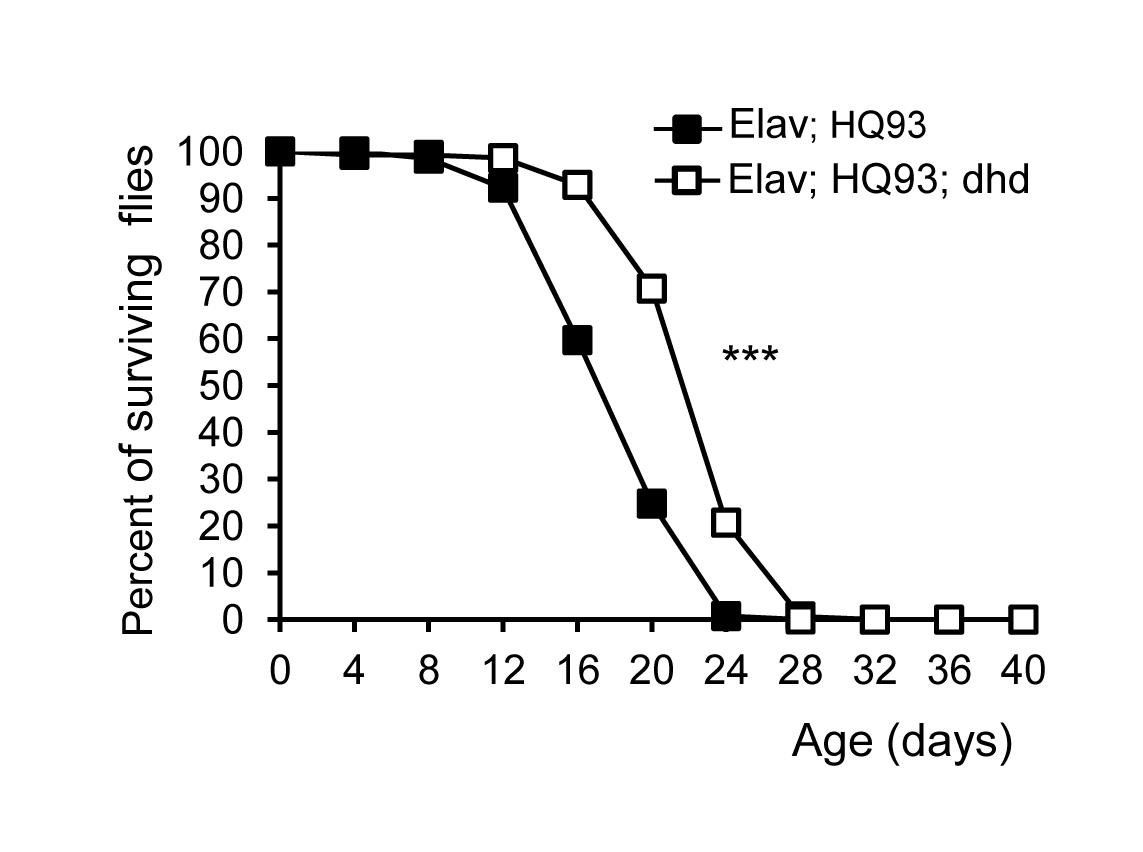

Supplement: S6 Fig — Lifespan of flies expressing the two transgenes dhd and HQ93 (open circles) was extended in comparison with control flies (filled triangles) with Elav-Gal4, n = 129 and 140 flies respectively. Survival curves were highly significantly different by log-rank test (***, p<0.0001). (TIF) [file pone.0118765.s006.tif]
