# Supplementary material for: Enhanced Neuronal Glucose Transporter Expression Reveals Metabolic Choice in a HD Drosophila Model
Source: PLoS One. 2015 Mar 11;10(3):e0118765. doi: 10.1371/journal.pone.0118765 (PMC4356621; doi:10.1371/journal.pone.0118765)
Supplement: S1 Table — (DOC) [file pone.0118765.s007.doc]

**S1 table : Control lines according to the genetic background of the *Drosophila*** lines used in experiments.

| **Génotype** | **Control line** |
| --- | --- |
| hGluT3 | yw |
| PFK | w^1118^ |
| G6PD | yw |
| Jafrac I | w^1118^ |
| deadhead | yw |
| PDH RNAi | w^1118^ |
| ND23 RNAi | w^1118^ |
