# Supplementary material for: Enhanced Neuronal Glucose Transporter Expression Reveals Metabolic Choice in a HD Drosophila Model
Source: PLoS One. 2015 Mar 11;10(3):e0118765. doi: 10.1371/journal.pone.0118765 (PMC4356621; doi:10.1371/journal.pone.0118765)
Supplement: S2 Table — (DOC) [file pone.0118765.s008.doc]

**S2** table : List of primers used for qPCR.

| Gene target | Forward sequence | Reverse sequence |
| --- | --- | --- |
| E1-PDH | TCGCGGCTTCTGTCATCTGTATTC | GATGCCATTGCCTCCGTAGAAGTT |
| ND23 | ACCGGCCACTATCAACTATCCCTT | TGACAGAATCCGCAGTAGATGCAC |
| rp49 | CACCAGTCGGATCGATATGCTAA | AATCTCCTTGCGCTTCTTGGAG |
